# Sane at Last

**Published:** 1915-12

**Authors:** 


### Sane at Last.

"It is one of nature's blunders, and I am willing that nature should correct its error by my baby's death." These were the words uttered by a mother of a hopelessly defective baby whose life might have been saved by an operation, and they mark the change in our regard for human sympathy, in our deeper understanding of social responsibility, and in eugenic progress. The occasion was the birth of a child in a Chicago hospital whose defects were such as to render it paralyzed and mentally irresponsible and physically deformed the rest of its life. Its intestinal tract was impervious, and an intestinal anastomosis would have been necessary to have saved its life. The physician in charge, Dr. H. J. Haiselden, took the position that a child so mentally and physically unfit should not have its life prolonged by surgical intervention. While the incident perhaps is not so unusual as to warrant such public notoriety, yet it has served the purpose of getting a public expression on a most important ethical question.

The unpardonable sin, the only one that nature knows, is the bringing into the world of a child mentally and physically unfit. But society has been so imbued with the idea of the sanctity of human life that the afflicted and defective have been protected and permitted to thrive as an act of humanity. The consequence has been the deterioration of the race. Nature, when let alone, cuts off the unfit that the fit might be preserved and the better to realize its purpose in life. So, when man assumed to transcend the prerogative of nature and permit the unfit to live, he must assume a further step of responsibility and control the procreation of the unfit. For, if the spirit of humanity demands the saving of human life whatever its condition, the same spirit demands its protection also. It is this responsibility that is the mission of eugenics. And, while we need not be less humanitarian, in our regard for human life, we must, nevertheless, be equally awake to the obligation we owe to the race and the unborn generations. It is not, therefore, a crime against the sanctity of life when the mental and physical defective are prevented from transmitting their degeneracy to future generations. And, further, in the light of this racial obligation, in such instances as above sighted, the doctor performed the highest moral duty in permitting the natural death of this child.

Thus we can exclaim, in the interest of the race, that we are at last becoming *sane*. We most heartily commend the doctor in his service to principle and his firm stand against his critics. We proudly hold this mother up as an example of true action in parental duty, even patriotism.

It is not sufficient to urge against such practice that environment has in some singular instance apparently overcome physical defects, for it is only apparent inasmuch as the hereditary defect of the recovered individual is transmitted to his offspring. We should remember that belief, whether true or false, is largely an inherited trait and is difficult to modify, though being a social heritage, it can be changed. This will explain why some people are so vehement in their opposition to eugenic form.

Let us hope that public sentiment is wholly aroused and that not only the necessity of preventing the progeny of defectives be realized, but that the same sanction will be granted in prohibiting procreation in cases of tuberculosis, grave heart lesions, syphilis and any other condition where the welfare of the mother requires it or the natural rights of the child will be violated.

In vindication of his position, Dr. Haiselden said: "It is our duty to defend ourselves and future generations against the mentally defective we allow to grow and suffer among us and add to our burden and our problems. All conscience says it is our duty.

"Farmers select the best stock for reproduction; the best seed without rust or other disease for sowing.

"Poor humans rely only upon chance, and defectives are as welcome as any to enlarge families. Think of it!

"And only the mother will look after the idiot child. If the mother is taken away, the father and brothers and sisters will not care for it. It is pitched into an institution forthwith, and there the burden of the poor unfortunate is weighed down even more.

"Institutions are an abomination. To herd the insane, or the lesser defectives like cattle, as is done, is a crime and a sin. And to make matters worse this unfortunate class has no attraction that is going to draw the keeper, the guard, the nurse or whoever takes care of it, to greater kindliness. Those who care for the insane, many of them, grow unfeeling, neglectful and indifferent.

"And yet I am asked to allow this child to live, knowing what I know. I feel toward one who would willingly allow a hydrophobic dog to run in a flock of sheep and do as impulse directed, because this one shrank from hurting or crossing the dog.

"If children of some of my critics were killed or injured by a defective, they would see a good reason for checking the supply of the latter in the world. I have talked to too many mothers of defectives to be swayed in my judgment in this case. Many of their tales are pitiable.

"One woman I know has a son who is quite subnormal. He is an excellent swimmer, and delights in the exercise. Once I said to her:

"'But suppose some time he should drown?' and the mother, with pitiful promptness, answered:

"'Wouldn't that be a blessing?'

"The mother did not pray for the boy's drowning, but at least did not hope against it.

"So let us be sensible. Let us approve the sterilization of the insane and the defective and of the children of habitual drunkards when both father and mother are so.

"Let us reproduce ourselves in 100 per cent fashion, so that by the weeding out of our undesirables we decrease their burdens and ours and lay the foundation for a normal race which would result four generations from now. Let us venerate a standard with soul and sense instead of desecrating it with crumbling tradition and mindless sentimentality."

---

Beginning with the January, 1916, number the *Journal of Cutaneous Diseases*, including syphilis, will be published for the American Dermatological Association by W. M. Leonard, of Boston. Each number will contain eighty pages, and as far as possible be of interest and value to the general practitioner as well as to the dermatologist.

---

Dr. Scurry Terrell, of Dallas, recently returned from France, where he was a medical officer of the British government for three and a half months. He says everybody goes to church in France now, and that in the fighting one man is killed for every three wounded, and in previous wars the rate was one killed for every ten wounded. He says a bomb dropped in a trench of 167 men killed 67 of them and wounded 78 of the others.

